# The Tropical Brown Alga *Lobophora variegata*: A Source of Antiprotozoal Compounds

**DOI:** 10.3390/md8041292

**Published:** 2010-04-16

**Authors:** Zulema Cantillo-Ciau, Rosa Moo-Puc, Leovigildo Quijano, Yolanda Freile-Pelegrín

**Affiliations:** 1 Department of Marine Resources, Cinvestav, Km 6 Carretera Antigua a Progreso, Cordemex, 97310, A.P. 73, Mérida, Yucatán, México; 2 Unidad de Investigación Médica Yucatán, Unidad Médica de Alta Especialidad, Centro Médico Ignacio García Téllez, Instituto Mexicano del Seguro Social, 41 No. 439 x 32 y 34, Col. Industrial, CP 97150, Mérida, Yucatán, México; E-Mail: moopuc@gmail.com; 3 Instituto de Química, Universidad Nacional Autónoma de México, Circuito Exterior, Ciudad Universitaria, Coyoacán, 04510 México City, México; E-Mail: quijano@servidor.unam.mx

**Keywords:** antiprotozoal activity, Lobophora variegata, sulfoquinovosyldiacylglycerol, structural characterization, algae

## Abstract

*Lobophora variegata*, a brown alga collected from the coast of the Yucatan peninsula, Mexico, was studied for antiprotozoal activity against *Giardia intestinalis*, *Entamoeba histolytica* and *Trichomonas vaginalis*. The whole extract showed the highest activity against *T. vaginalis*, with an IC_50_ value of 3.2 μg/mL. For the fractions, the best antiprotozoal activity was found in non-polar fractions. The chloroform fraction of the extract contained a major sulfoquinovosyldiacylglycerol (SQDG), identified as 1-*O*-palmitoyl-2-*O*-myristoyl-3-*O*-(6‴-sulfo-α-d-quinovopyranosyl)-glycerol (**1**), together with small amounts of 1,2-di-*O*-palmitoyl-3-*O*-(6‴-sulfo-α-d-quinovopyranosyl)-glycerol (**2**) and a new compound identified as 1-*O*-palmitoyl-2-*O*-oleoyl-3-*O*-(6‴-sulfo-α-d-quinovopyranosyl)-glycerol (**3**). Their structures were elucidated on the basis of chemical and enzymatic hydrolysis and careful analysis of FAB-MS and NMR spectroscopic data. This is the first report on the isolation of SQDGs from *L. variegata*. The mixture of **1**–**3** showed good activity against *E. histolytica* and moderate activity against *T. vaginalis* with IC_50s_ of 3.9 and 8.0 μg/mL, respectively, however, the activity of **1**–**3** is not as effective as metronidazole. These results afford ground information for the potential use of the whole extract and fractions of this species in protozoal infections.

## 1. Introduction

The three anaerobic protozoa, *Entamoeba histolytica*, *Giardia intestinalis* (commonly referred to as *Giardia lamblia*) and *Trichomonas vaginalis* are highly prevalent human-infective parasites with a worldwide distribution, which despite their significant differences in life-cycle and pathogenic properties are customarily grouped together based on their carbohydrate metabolism and their lack of mitochondria [1]. Enteric protozoan infections produced by *G. lamblia* and *E. histolytica* are a significant cause of morbidity and mortality in developing countries. Symptomatic patients typically present diarrhea and abdominal symptoms, including stomachache, cramps, bloating or tenderness. *Entamoeba histolytica* is estimated to cause severe disease in 48 million people, killing 70,000 each year [2]. Although *G. intestinalis* rarely causes a lethal disease, it is the most common intestinal parasite of humans in developed countries. In Asia, Africa and Latin America, about 200 million people have symptomatic giardiasis, with about 500,000 new cases reported each year [3]. On the other hand, Trichomoniasis is a common sexual transmitted disease caused by *T. vaginalis* which infects the urogenital tract of men and women. Worldwide, it is responsible for the annual infection of 180 million people [4]. This has been associated with vaginitis, cervicitis, urethritis, prostatitis, epididymitis, cervical cancer, infertility and pelvic inflammatory disease [5]. Currently, the drug of choice for treating these infections is metronidazole, a 5-nitroimidazole derivative that possesses activity against anaerobic microorganisms [6]. However, resistance to metronidazole has been demonstrated in these protozoans, both in natural and experimental conditions [7]. Also, metronidazole is effective with prolonged treatment or high doses and can produce many side effects including headache, nausea, gastrointestinal disturbance, anorexia and dizziness [8]. Recent studies have also demonstrated cytotoxic effects of metronidazole [9]. Therefore, the search for more efficient and safer antiprotozoal agents is justified.

Throughout history the plant kingdom has provided a variety of medicines. It is noticeable that most of the studies have focused on natural substances derived from higher plants, mainly due to their accessibility. Nevertheless, the metabolic and physiological capabilities of marine organisms that allow them to survive in complex habitat provide a great potential for production of secondary metabolites, which are not found in terrestrial environments. Thus, marine algae are among the richest sources of known and novel bioactive compounds [10,11]. In the course of our ongoing investigations toward the isolation of bioactive metabolites from marine algae of the Yucatan coast (Mexico), we recently studied the bioactivity of several algal species belonging to the three algae divisions: Phaeophyta, Chlorophyta and Rhodophyta (brown, green and red algae respectively). The results showed that the brown alga *Lobophora variegata* has high antiparasitic activity [12,13] as well as antioxidant activity [14]. Therefore, *L. variegata* was selected for fractionation and isolation of its antiprotozoal constituents. In this paper we report the antiprotozoal activity of the whole extract and fractions from *L. variegata*, using *in vitro* antiprotozoal assay, as well as the isolation of three sulfoquinovosyl-diacylglycerols **1**–**3** (SQDGs **1**–**3**) from the chloroform fraction of the total extract with antiprotozoal activity. The cytotoxicity of the whole extract, fractions and SQDGs **1**–**3** on mammalian cells was also assessed in order to provide selective index for further studies.

## 2. Results and Discussion

**Figure 1:**
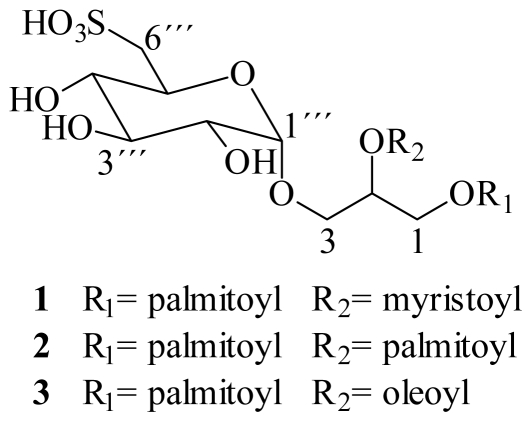
Structures of SQDG’s **1**–**3** isolated from *Lobophora variegata.*

The dried and powered alga *Lobophora variegata* was extracted with 7:3 dichloromethane-methanol and concentrated at 45 °C. The resulting dried extract was dissolved in methanol-water (9:1) and successively partitioned against hexane, chloroform, ethyl acetate and *n*-butanol, following a modified Kupchan procedure [15]. The chloroform fraction showing the best antiprotozoal activity against the three protozoa *G. intestinalis*, *E. histolytica* and *T. vaginalis* (see Table 3) was chosen for further fractionation and isolation of its components. Chromatographic fractionation on Sephadex LH-20, allowed the isolation of a mixture of SQDGs **1**–**3** (Figure 1). The mixture of SQDGs was colorless amorphous powder, apparently homogenous on TLC, yielding purplish pink spots on spraying with methanolic sulfuric acid.

Careful analysis of the ^1^H- and ^13^C-NMR data including ^1^H-^1^H COSY, TOCSY, DEPT, HSQC and HMQC spectra, allowed the assignment of all ^13^C- and ^1^H-NMR signals (Table 1). Three spin systems were recognized, the first one was assigned to a glycerol moiety [δ_H_ 4.34 and 4.13 (δ_C_ 62.7); δ_H_ 5.13 (δ_C_ 69.8); δ_H_ 3.89 and 3.39 (δ_C_ 64.6)].

The cross-peaks in the HMBC spectrum [δ_H_/δ_C_: 5.13 (H*sn*-2)/172.4, 172.6 (COO); 4.34 and 4.13 (H*sn*-1)/172.4, 172.6 (COO); 2.28 (α-CH_2_)/172.4, 172.6 (COO)] indicated the presence of acyl groups on the *sn*-1 and *sn*-2 positions of the glycerol moiety. Therefore, the second spin system signals were attributable to two fatty acyl groups whose terminal methyl signals appeared overlapped at δ_H_ 0.84 (6H, t, *J* = 6.9 Hz, δ_C_ 14.0).

The third spin system indicated the presence of a glycosyl moiety. Combination of TOCSY and COSY data allowed the identification of a sequence of four oxymethynes and one deshielded methylene. Hence, taking the anomeric proton at δ_H_ 4.57 (δ_C_ 98.3) as a safe entry, this proton showed cross-peaks with signals at δ_H_ 2.92, 3.18 and 3.35 in the TOCSY spectrum.

Similarly, the methylene protons at δ_H_ 2.90 and 2.54 showed cross-peaks with the same signals, besides the signal at δ_H_ 3.77, indicating that all these signals belongs to an hexapyranose with the substitution pattern and relative stereochemistry similar to glucose. Then, COSY spectrum allowed unambiguous assignment of all protons of the glycosyl moiety (see Table 1).

Confirmation of the linkage of the glycosyl moiety at *sn*-3 of glycerol, was based on the HMBC cross-peak between the anomeric proton (H-1‴, δ_H_ 4.57) and carbon C-3 at δ_C_ 64.6. The relative small coupling constant value of the anomeric proton (H-1‴), *J* = 3.5 Hz, indicated the α orientation of the glycosidic union, while the large vicinal coupling constants observed between H-2‴/H-3‴, H-3‴/H-4‴ and H-4‴/H-5‴ (*J* = 9.6 Hz), indicated the glucopyranosyl nature of the sugar moiety. On the other hand, the ^1^H- and ^13^C-NMR characteristic chemical shifts of the methylene protons, H-6‴ (δ_H_ 2.90 and 2.54) and carbon C-6‴(δ_C_ 54.5) indicated the presence of a sulphonyl group attach at C-6‴ carbon, of the sugar, instead of the hydroxyl group of glucose [16]. The presence of a small triplet at δ_H_ 5.31 (*J* = 4.7 Hz) due to olefinic protons in the ^1^H-NMR, spectrum suggested the presence of small amounts of another compound with an unsaturated fatty acid (Table 1).

All proton and carbon NMR data are in good agreement with the presence of 6-deoxy-6-sulpho-α-d-glucopyranosyl-1,2-*O*-diacyl-glycerols. To identify the acyl substituents at *sn*-1 and *sn*-2 in the mixture, enzymatic and alkaline hydrolysis were performed. It is known that the enzymatic hydrolysis using Lipase from *Mucor javanicus*, regioselectively liberate the acyl moiety at *sn*-1 position [17]. The GC-MS analysis of the enzymatic hydrolysis product indicated the presence of palmitic acid only. Thus, it was concluded that the palmitoyl residue was attached to *sn*-1 position of the glycerol moiety in the mixture.

On the other hand, to infer the exact nature of the fatty acids in *sn*-2 position, the mixture was subjected to alkaline hydrolysis with NaOMe in MeOH. After partitioning, the organic extract was analyzed by GC/MS, the composition of the fatty acid methyl esters was shown to be methyl myristate, methyl oleate and methyl palmitate, being the last in greater proportion.

**Figure 2:**
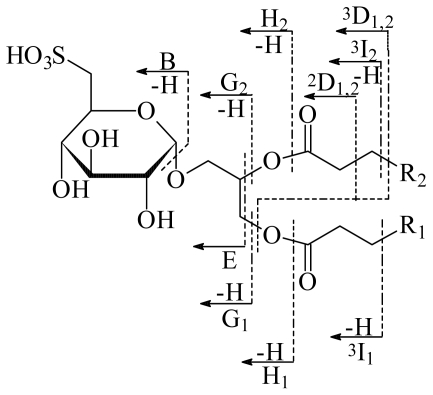
Nomenclature for cleavage of glycolipids and some fragmentation scheme of SQDG [18]. For the cleavage of fatty acyl chains the subscript in the symbol represents the relative position (*sn*-1 or *sn*-2) of the cleavage in the fatty acyl chain and the superscript the cleaved bond position relative to the carbonyl carbon of the fatty acyl group. In the proposed positive ions there are two sodium ions attached at the terminal sulfonic group.

In this study, the mass spectra of the mixture of SQDGs were obtained by FAB in negative and positive ion modes. Careful analysis of the spectra allowed the identification of three compounds. The diagnostic ion peaks [M-H + 2Na]^+^ (at m/z 811, 839 and 865) appearing in the positive-ion FAB spectrum and [M-H]^−^ (at m/z 766, 794 and 820) in the negative-ion FAB spectrum. Confirmation of the double sodium adduct was determined by HR-FAB-MS, where the peak at m/z 811 is in accordance with the formula of C_39_H_73_O_12_SNa_2_ (*m/z* 811.4614, C_39_H_73_O_12_SNa_2_, requires 811.4618). The presence of [M-H+2Na]^+^ instead of [M+Na]^+^ in the positive-ion FAB spectrum is due to the acidity of the sulfonic acid group at position 6 of the sugar unit [18]. Namely, the positive charge is located at the terminal sulfonic group. This is further supported by the presence of a prominent peak at m/z 126 in the positive-ion FAB spectrum, which can be assigned to the [SO_3_Na_2_]^+^ ion. The nomenclature for cleavages of SQDG proposed by Costello and co-workers is adopted in this paper [19,20]. Thus, we can divide the fragmentation ions into two types, one generated by the cleavage of the sugar moiety and the other by the cleavage of the fatty acyl chains (Figure 2). All the ions observed in the positive-ion FAB spectrum correspond to sodium-attached ions. The cleavage of the interglycosidic bond results in the ion peak at m/z 271. Two ions resulting from the concurrent cleavage of the bond β or γ to the carbonyl group of one fatty acyl chain and the cleavage of the ester bond of the other fatty acyl chain are observed at m/z 387 (^2^D_1,2_) and 401 (^3^D_1,2_), respectively. The other ions observed are mainly produced by the fragmentation of fatty acyl groups. The loss of the acyl groups as the corresponding fatty acid (-RCOOH) can be observed as the ions G_1_ and G_2_. For SQDG’s **1**–**3** three ions G_1_ resulting from the loss of palmitic acid at *sn*-1 position are observed at m/z 555, 583 and 609 respectively. One only ion G_2_ is observed for the three compounds and is produced by the loss of myristic, palmitic and oleic acids respectively at position *sn*-2. Other ions that supported the presence of three SQDGs can be observed in Figure 2 and Table 2.

On the basis of the molecular formula obtained by high resolution positive FAB MS, ^1^H- and ^13^C-NMR data and the results from the selective hydrolysis, the structure of compound **1** was established as 1-*O*-palmitoyl-2-*O*-myristoyl-3-*O*-(6‴-sulfo-α-d-quinovopyranosyl)-glycerol, while compounds **2** and **3** were identified as 1,2-di-*O*-palmitoyl-3-*O*-(6‴-sulfo-α-d-quinovopyranosyl)-glycerol and 1-*O*-palmitoyl-2-*O*-oleoyl-3-*O*-(6‴-sulfo-α-d-quinovopyranosyl)-glycerol, respectively.

Concerning the stereochemistry at C-2 in the glycerol portion, it was tentatively assumed to be S, based on comparison of the NMR data and optical rotation of **1**–**3** ([α]^25^_D_ + 43.18 in MeOH, *c* 0.44) with those of the SQDGs isolated from *Byrsonima crassifolia* [21]. Furthermore, most of natural glycoglycerolipids, including SQDGs of known absolute stereochemistry have S-configuration (*sn*-1,2-diacylglycerols) [22]. As for the quinovose (6-deoxyglucose) moiety, it was assumed to have the d-configuration, based on the common configuration of glucose in nature [23].

**Figure 3:**
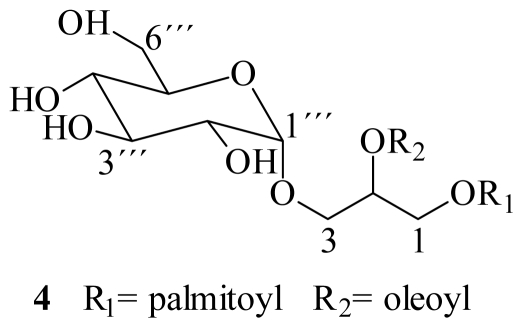
Glucosyldiacylglycerol isolated from *Sargassum fulvellum*.

Shao *et al*. [24], reported the isolation of the sulfoglycolipid crassicaulisine, with myristoyl and palmitoyl as acyl groups at positions 1 and 2 of glycerol respectively, from the red alga *Chondria crassicaulis*. Compound **1**, the major SQDG of *L. variegata*, corresponds to the positional isomer of crassicaulisine, where palmitoyl and miristoyl acyl groups are interchanged, *i.e.*, at *sn*-1 and *sn*-2 respectively. There is a solitary reference in the literature on the identification of **1** from the brown alga *Ishige okamurai* [25]. In the case of compound **2**, it has been isolated from several terrestrial and marine sources, as from leaves of *Byrsonima crassifolia* [21,26], from the brown seaweeds *Ishige okamurai* and *Sargassum parvivesiculosum* [25,27], from the green algae *Caulerpa racemosa* and *Dictyochloris fragrans* with notable selective antiviral activity against HSV-2 and a P-selectin receptor inhibitor [16,28]. Concerning compound **3**, there are no reports of its isolation from natural sources; therefore it corresponds to a new natural product. Recently, the isolation of the glucosyldiacylglycerol **4**, from the brown alga *Sargassum fulvellum*, was published (Figure 3). The proposed structure differing from **3** only in the presence of a glucosyl moiety, instead of the sulfonic derivative at C-6‴ [29]. However, it is noteworthy the chemical shift reported for C-6‴ of the glucose at δ_C_ 54.9, which is characteristic value for C-6‴ in sulfoglycolypids or aminoglycolipids. Therefore, the structure **4** should be revised.

Previous studies have reported extensive biological effects for SQDG, such as DNA polymerase inhibition, immunosuppressive effect, human immunodeficiency virus reverse transcriptase inhibition, antimicrobial and anti-tumor activities [30–35]. In this study, the evaluation of the whole extract (dichloromethane-methanol) and its partition fractions (hexane, chloroform, ethyl acetate, *n*-butanol and aqueous residue) against *G. intestinalis*, *E. histolytica* and *T. vaginalis*, was carried out. Due to the lack of data on antiprotozoal activity scores from natural product extracts, we define high activity as an IC_50_ ≤ 5 μg/mL, moderate activity as IC_50_ 5.1–10 μg/mL and low activity as IC_50_ > 10 μg/mL [12]. The cytotoxic effect on one normal cell line (MCDK) was determined using the MTT assay. In order to evaluate the level of harmfulness of the substances on normal cells, the selectivity index (SI) of each extract and fractions was also determined, it is generally considered that biological efficacy is not due to cytotoxicity when SI ≥ 10 [36].

The antiprotozoal activity (IC_50_) of the whole extract and its fractions are shown in Table 3. The whole extract of *Lobophora* showed high activity against *T. vaginalis* with IC_50_ value of 3.2 μg/mL and low activity against *G. intestinalis* and *E. histolytica* (IC_50_ 10.5 and 10.8 μg/mL respectively). When the whole extract was fractionated by polarity, fractions showed variable activity against the protozoa.

In case of *Giardia intestinalis*, the chloroform fraction was the most active (0.5 μg/mL), but also the activity of hexane and ethyl acetate fractions were very good (1.0 and 0.8 μg/mL, respectively). The activity of chloroform fraction is close to that of metronidazole (drug used as the positive control) and the selectivity index is adequate (SI = 175). Only the *n*-butanol fraction was not active.

In case of *Entamoeba histolytica*, the ethyl acetate fraction was the most active with an IC_50_ of 1.7 μg/mL (SI = 294.2). The activity of hexane fraction, however, was also important, with an IC_50_ of 2.8 μg/mL. *T. vaginalis* trophozoites were sensible to whole extract and chloroform fraction (3.2 and 3.7 μg/mL, respectively), while the *n*-butanol fraction and aqueous residue were inactive.

For fractions, the best antiprotozoal activity was found in non-polar fractions. From these, the chloroform fraction showed the best activity against the three protozoa with good selectivity (SI > 10), therefore, it was chosen for the isolation of active compounds.

The mixture of SQDGs **1**–**3** isolated from the chloroform fraction was tested for antiprotozoal activity. The mixture showed high *in vitro* activity against *E. histolytica* (IC_50_ 3.9 μg/mL) and moderate activity against *T. vaginalis* trophozoites (IC_50_ 8 μg/mL) with good selective index (SI > 10) (Table 3) however, the activity of **1**–**3** is not as effective as metronidazole. In addition, the chloroform fraction showed the stronger activity than the purified compounds against *G. intestinalis* and *T. vaginalis*. This could be attributed that the SQDGs are not the major inhibitory compounds on the parasites growth and that the inhibitory effects are attributed to other unidentified compounds in the same fraction and/or occurrence of synergistic activities between different components within a fraction. These results show the possibility that crude extract and non-polar fractions from *L. variegata* could be used rather than the purified SQDGs for development of new antiprotozoal drugs. In this sense, toxicity and efficacy *in vivo* studies are now in progress, also, the chemical study of ethyl acetate fraction are currently on-going.

## 3. Experimental Section

### 3.1. General experimental procedures

Optical rotations were measured on a Perkin-Elmer model 383 digital polarimeter. NMR spectra were recorded in CD_3_SOCD_3_, on a Bruker Avance 400 spectrometer. The chemical shifts are given in δ (ppm) with residual CD_3_SOCD_3_ as internal reference and coupling constants in Hz. FABMS were recorded with a NBA matrix in the positive and negative modes, on a JEOL JMS-SX 102A. Precoated TLC silica gel 60 F_254_ aluminum sheets from Merck were used for analytical thin-layer chromatography (0.25 mm layer thickness) and the compounds were visualized as purplish spots on spraying with 5% methanolic sulfuric acid followed by heating at 100 °C. Solvent system for TLC was dichloromethane-methanol (85:15). Column chromatography was conducting using Sephadex LH20 from Sigma-Aldrich.

### 3.2. Algal material

The brown algae *Lobophora variegata* (J.V. Lamouroux) Womersley ex E.C. Oliveira (Phaeophyta) was collected in March 2007 in Puerto Morelos (20°46′N–88°05′W). Once harvested, the alga was stored in plastic bags and chilled on ice during transport to the laboratory. A voucher specimen was identified according to Littler and Littler [37] and deposited at the Cinvestav Marine Algae Herbarium.

### 3.3. Extraction and isolation of **1**–**3**

Collected samples of *Lobophora variegata* were washed thoroughly with freshwater to remove salts, sand and epiphytes, and stored at −20 °C. Entire plants were lyophilized and milled into powder (800 g) and were thoroughly extracted with dichloromethane-methanol (7:3) at room temperature. The solvent was removed by distillation at 45 °C to give 35.5 g of crude extract residue. The crude residue was dissolved in methanol-water (9:1) and successively subjected to solvent partitioning according to Kupchan’s modified method [15] using, hexane, chloroform, ethyl acetate and *n*-butanol. Five fractions of different polarity, including the aqueous one, were obtained. The solvent was eliminated by distillation *in vacuo*, yielding 10.0, 3.6, 16.7, 3.4 and 1.8 g of residue, respectively. The chloroform fraction was subjected to column chromatography on Sephadex LH20, eluted with hexane-chloroform-methanol 3:2:1. A total of 25 fractions (25 mL/each fraction) were collected. Fraction 24 was concentrated, precipitated with acetone and filtered to give a mixture of **1**–**3** (276.3 mg).

### 3.4. Alkaline hydrolysis

A solution of **1**–**3** (6 mg) in MeOH (0.5 mL) was treated with NaOMe 0.5M in MeOH (1 mL) and stirred at room temperature for 5 h. After this time, the reaction mixture was neutralized with Dowex 50W × 4 and the resin removed by filtration. The filtrate was extracted with hexane and the hexane layer was concentrated and analyzed by GC-MS: Agilent Technologies 6890N GC equipped with a mass-selective detector 5973Network MSD, a split injector and a silica-capillary GC column HP-5MS (30 m × 0.25 mm; i.d. 0.25 μm film, Agilent Technologies, Inc.); the column oven temperature was kept at 80 °C for 1 min and then increased at a rate of 15 °C/min up to 310 °C, carrier He, flow rate 0.8 mL/min. Methyl palmitate, methyl myristate and methyl oleate were detected with retention times of 9.64, 11.05 and 12.16 min, respectively. Their retention times and fragmentation peaks were in good agreement with those of standard fatty methyl esters.

### 3.5. Enzymatic hydrolysis

The mixture of **1**–**3** (0.3 mg) was mixed with lipase (1 mg) from *Mucor javanicus* (Sigma-Aldrich, Switzerland, Ec 2326199) in dioxane-H_2_O (1:1, 1 mL) and the reaction mixture was incubated at 38 °C for 4 h. After removal of the dioxane and H_2_O, the residue was dissolved in 2 mL MeOH and partitioned with hexane. The hexane extract was analyzed by GC-MS. Palmitic acid was detected with retention time of 11.11 min. Retention time was identical to those of the authentic standard.

### 3.6. Antiprotozoal activity

*Trichomonas vaginalis* and *Entamoeba histolytica* strain GT3 and HM1: IMSS respectively were maintained in TYI-S- 33 medium supplemented with10% bovine serum. *Giardia intestinalis* strain IMSS:0696:1 were grown in TYI-S-33 modified medium, supplemented with 10% calf Serum. For the assays trophozoites were axenically maintained and employed in the log phase of growth.

*In vitro* susceptibility assays used the sub-culture method of Cedillo-Rivera *et al*. [38]. The extracts were dissolved in 1 mL of dimethylsulfoxide (DMSO) and added to microtubes containing 1.5 mL of medium in order to reach concentrations of 1.6, 3.3, 6.6, and 13.3 μg/mL. The solutions were inoculated with trophozoites to achieve an inoculum 1 × 10^4^, 2 × 10^4^ and 6 × 10^3^ trophozoites/mL of *T. vaginalis*, *G. lamblia and E. histolytica*, respectively and then incubated for 48 h at 37 °C. Each test included metronidazole as standard drug, a control (culture medium plus trophozoites and DMSO), and a blank (culture medium). After incubation, the trophozoites were detached by chilling and samples of each tube were sub-cultured in fresh medium for another 48 h, without antiprotozoal drugs or extracts. The final number of parasites was determined with a haemocytometer and the 50% inhibitory concentration (IC_50_) was calculated by Probit analysis. The concentrations were performed twice in each experiment and the experiments were performed by triplicate

### 3.7. Cytotoxicity assay

Madin-Darby canine kidney cell (MDCK) were grown in DMEM (Gibco) media supplemented with 10% (v/v) fetal bovine serum (FBS;Gibco) with 100 U/mL penicillin and 100 μg/mL streptomycin and maintained at 37 °C in a 5% CO_2_ atmosphere with 95% humidity.

Cytotoxicity assays with the crude extract, each one of the fractions (A-E) and the mixture of **1**–**3**, were performed according to Rahman *et al*. [39]. Briefly, 1.5 × 10^4^ MDCK viable cells were seeded in a 96-well plate (Costar) and incubated for 24–48 h. When reached >80% confluence, the medium was replaced and the cells were treated with the crude extracts at 6.25, 12.5, 25 and 50 μg/mL dissolved in dimethylsulfoxide (DMSO) at a maximum concentration of 0.05%. After 72 h of incubation, the cells were treated as described in Moo-Puc *et al.*, [12]. Metronidazole was used as positive control, whereas untreated cells were used as negative controls. The concentration of the substances that killed 50% of the cells (CC_50%)_ was calculated by GraphPad Prim 4 software. The concentrations were performed twice in each experiment and the experiments were performed by triplicate.

The selective index (SI) was defined as the ratio of cytotoxicity to biological activity (SI = CC_50_ MDCK cells/IC_50_ protozoa) was calculated.

## 4. Conclusions

The tropical brown alga *Lobophora variegata* collected from the coast of the Yucatán peninsula, México, is a source of antiprotozoal compounds. The whole extract and non-polar fractions from this species could be used for development of new antiprotozoal drugs. A mixture of sulfoquinovosyldiacylglicerols, SQDGs **1**–**3** was isolated from the chloroform soluble fraction of whole extract of the alga and it comprises a major SQDG, identified as 1-*O*-palmitoyl-2-*O*-myristoyl-3-*O*-(6‴-sulfo-α-d-quinovopyranosyl)-glycerol (**1**), together with small amounts of 1,2-di-*O*-palmitoyl-3-*O*-(6‴-sulfo-α-d-quinovopyranosyl)-glycerol (**2**) and a new compound identified as 1-*O*-palmitoyl-2-*O*-oleoyl-3-*O*-(6‴-sulfo-α-d-quinovopyranosyl)-glycerol (**3**). This is the first report on the presence of the SQDGs **1**–**3**, in *Lobophora variegata* species. Moreover, the activity against *G. lamblia* and *E. histolytica* of whole extract and fractions of *L. variegata* was also revealed for the first time in this study.

## Figures and Tables

**Table 1:** NMR spectroscopic data for mixture of **1**–**3**.

	Compounds 1 and 2		Compound 3	

position	δ_H_/mult., (*J* in Hz)	δ_C_	δ_H_/mult., (*J* in Hz)	δ_C_
1a	4.34 dd (12.0, 2.4)	62.7	4.34 dd (12.0, 2.4)	62.7
1b	4.13 dd (12.0, 7.5)		4.13 dd (12.0, 7.5)	
2	5.13 m	69.8	5.13 m	69.8
3a	3.89 dd (10.5, 6.0)	64.6	3.89 dd (10.5, 6.0)	64.6
3b	3.39		3.39	
1‴	4.57 d (3.5)	98.3	4.57 d (3.5)	98.3
2‴	3.18 dd (3.5, 9.6)	71.7	3.18 dd (3.5, 9.6)	71.7
3‴	3.35	72.9	3.35	72.9
4‴	2.92 dd (9.6)	74.2	2.92 dd (9.6)	74.2
5‴	3.77 ddd (9.6, 6.8, 4.3)	68.6	3.77 ddd (9.6, 6.8, 4.3)	68.6
6‴a	2.90 dd (14.0, 4.3)	54.5	2.90 dd (14.0, 4.3)	54.5
6‴b	2.54 dd (14.0, 6.9)		2.54 dd (14.0, 6.9)	
1′, 1″		172.4, 172.6		172.4, 172.6
2′, 2″	2.28 m	33.6	2.28 m	33.6
3′, 3″	1.49 m	31.4	1.49 m	31.4
8″, 11″			1.97 m	26.7
9″, 10″			5.3 t (4.7)	129.61, 129.64
-CH_2_	1.22 bs	29.2–22.2	1.22 bs	29.2–22.2
CH_3_	0.84 t (6.9)	14.0	0.84 t (6.9)	14.0

Recorded in DMSO-*d*_6_ at 400 MHz; chemical shifts, multiplicity and coupling constants (*J*, Hz) were assigned by means of ^1^H-, ^13^C-NMR and 2D NMR data.

**Table 2:** Fragmentation assignment of product ions observed in positive mode FAB-MS of sodium adducted molecular ions of the mixture of SQDGs **1**–**3**.

	Product ions, m/z		

Assignment	SQDG-1 [M-H+2Na]^+^ = 811	SQDG-2 [M-H+2Na]^+^ = 839	SQDG-3 [M-H+2Na]^+^ = 865
B	271	271	271
E	329	329	329
^2^D_1,2_	387	387	387
^3^D_1,2_	401	401	401
G_1_	555	583	609
G_2_	583	583	583
H_1_	571	599	625
H_2_	599	599	599
^3^I_1_	627	655	681
^3^I_2_	655	655	655
SO_3_Na_2_^+^	126	126	126

**Table 3:** Antiprotozoal activity, cytotoxic activity on Madin-Darby canine kidney cells and selectivity index of whole extract, fractions and compounds from *Lobophora variegata*.

Extract/fraction/compound (weight)	Antiprotozoal activity IC_50_ μg/mL			Cytotoxicity CC_50_ μg/mL	Selectivity index SI		
	Gi	Eh	Tv	MDCK	MDCK/Gi	MDCK/Eh	MDCK/Tv
Whole extract (35.5 g)	10.5 ± 0.45	10.8± 0.09	3.2± 0.09	78.0 ± 2.45	7.4	7.2	24.4
Hexane fraction (10 g)	1.0 ± 0.06	2.8 ± 0.08	13.4 ± 0.61	65.0 ± 1,23	65	23.2	4.9
Chloroform fraction (3.6 g)	0.5 ± 0.02	6.2 ± 0.32	3.7 ± 0.11	87.5 ± 0.89	175	14.1	23.6
Ethyl acetate fraction (16.7 g)	0.8 ± 0.03	1.7 ± 0.04	11.7 ± 0.22	500.1 ± 3.4	625.1	294.2	42.7
*n*-butanol fraction (3.4 g)	-	15.5 ± 0.73	-	90.8 ± 1.78	nt	5.9	nt
Aqueous residue (1.8 g)	8.8 ± 0.71	15.6 ± 0.41	-	789.9 ± 2.67	89.8	50.6	nt
SQDG’s **1**–**3** (276.3 mg)	20.9 ± 0.89	3.9 ± 0.03	8.0 ± 0.42	85.3 ± 1.26	4.1	21.9	10.7
Metronidazole	0.22 ± 0.0005	0.13 ± 0.0005	0.04 ± 0.02	68 ± 1.2	309	523	1700

Gi: *Giardia intestinalis*; Eh: *Entamoeba histolytica*; Tv: *Trichomonas vaginalis*; - inactive >50 μg/mL; nt: not tested.
